# Concomitant age old infections of mankind – tuberculosis and leprosy: a case report

**DOI:** 10.1186/1752-1947-1-43

**Published:** 2007-07-05

**Authors:** Chandrashekhar T Sreeramareddy, Ritesh G Menezes, PV Kishore

**Affiliations:** 1 Department of Community Medicine, Manipal Teaching Hospital, Manipal College of Medical Sciences, Pokhara, Nepal; 2 Department of Forensic Medicine, Manipal Teaching Hospital, Manipal College of Medical Sciences, Pokhara, Nepal; 3 Department of Internal Medicine, Manipal Teaching Hospital, Manipal College of Medical Sciences, Pokhara, Nepal

## Abstract

**Background:**

Concomitant leprosy and tuberculosis is reported infrequently. Literature suggests that the infrequent occurrence of both leprosy and tuberculosis is based on the transmission dynamics of both the infections.

**Case presentation:**

Two cases of pulmonary tuberculosis diagnosed in patients being treated with glucocorticoids for complications of leprosy are reported.

**Conclusion:**

Clinicians should recognize corticosteroid treated leprosy patients as a population likely to develop concomitant tuberculosis.

## Background

There have been sporadic reports of co-existence of tuberculosis and leprosy in the same patients [1-4]. These reports suggest that it is important to identify these cases to avoid monotherapy of tuberculosis. One study has also reported that tuberculosis may occur through the spectrum of leprosy [5]. We report two cases of leprosy who were on treatment for related complications and were also diagnosed with pulmonary tuberculosis in our teaching hospital.

## Case presentation

### Patient 1

A 65-year-old non-hypertensive, non-diabetic man presented with history of left sided chest pain associated with productive cough and breathlessness for seven days. There was no history of fever, haemoptysis and exertional increase in breathlessness. The patient was referred to our teaching hospital from Green Pastures Hospital (GPH), a tertiary care referral centre for leprosy. The patient was treated for leprosy with WHO multidrug therapy for multibacillary leprosy. The patient was currently on treatment for ulnar neuritis with prednisolone 30 mg once a day for last three months. There was no past history of tuberculosis as per GPH records or information provided by the patient.

Clinical examination revealed numerous hypopigmented skin lesions varying in size from 2 to 10 centimeters over the trunk. The left ulnar nerve was thickened. There was dullness and decreased breath sounds in the left infrascapular and axillary areas.

The laboratory investigations revealed hemoglobin: 14.4 mg/dl; total leukocyte count: 19.5×10^3^; erythrocyte sedimentation rate: 60 mm at the end of first hour; serum creatinine: 1.4 mg/dl; and random blood sugar: 91 mg/dl.

One out of three sputum samples tested was positive for acid fast bacilli and the chest radiograph showed loculated pleural effusion suggesting long standing pleural collection in left hemi thorax with infiltrations bilaterally, more on left side.

A diagnosis of borderline leprosy with concomitant pulmonary tuberculosis was made. The patient was referred to DOTS clinic of our teaching hospital and started on category-I treatment according to the guidelines of national tuberculosis programme of Nepal. The oral prednisolone was subsequently tapered and stopped. The patient's general condition improved and was on regular follow up.

### Patient 2

A 50-year-old non-diabetic, non-hypertensive man who had completed treatment for lepromatous leprosy two years back, was admitted at Green Pastures Hospital for type-2 lepra reaction (erythema nodosum leprosum). There was a past history of lepra reactions for which he underwent treatment. Currently the patient was again being treated for the same with prednisolone 30 mg once daily, thalidomide 100 mg twice daily and clofazimine 50 mg once daily. However, information about initial screening of tuberculosis was not available.

The patient was referred to our teaching hospital with complaints of fever, productive cough and dyspnoea of three months duration. The patient also complained of pain and distension of abdomen and decreased appetite for three months.

Clinical examination revealed tense abdomen with shifting dullness, respiratory system with bilateral crackles with bronchial breath sounds predominantly in the left infra clavicular area, and skin showed no active lesions of erythema nodosum leprosum. The laboratory investigations revealed hemoglobin: 11.8 mg/dl; total leukocyte count: 5900/mm^3^; erythrocyte sedimentation rate: 110 mm in first hour; random blood sugar: 109 mg/dl; serum electrolytes, sodium: 135 meq/L and potassium: 3.2 meq/L.

Two out of three sputum samples were positive for acid fast bacilli. Chest radiograph showed multiple heterogeneous opacities in bilateral lung fields and a cavitary lesion in left upper zone. A diagnosis of pulmonary tuberculosis and provisional diagnosis of peritoneal tuberculosis were made.

Dermatologist's opinion was sought for the lepra reaction. The patient was advised to stop prednisolone as no active lesions persisted. He was subsequently referred to DOTS clinic and was started on category-I treatment.

## Discussion

Literature suggests that a cross-immunity exists between tuberculosis and leprosy. Chaussinad was the first to observe that in several different settings the prevalence of leprosy was inversely related to that of tuberculosis. Therefore, it may be unusual to come across co-existence of tuberculosis and leprosy. A study from South Africa reported that there was an increased incidence of pulmonary tuberculosis among leprosy patients but not vice versa [6]. Researchers have also suggested that individuals acquired protection against leprosy by previous infection/exposure to tuberculosis. Therefore, it is believed that BCG vaccine may give some protection against leprosy [7]. This theory of cross-immunity between tuberculosis and leprosy lead to the hypotheses for disappearance of leprosy from Western Europe before the advent of chemotherapy [8]. A study based on examination of DNA by PCR technique investigated the relation between tuberculosis and leprosy among archaeological samples. Researchers found the evidence for co-existence of tuberculosis and leprosy [9]. This supports an earlier report that tuberculosis can occur throughout the spectrum of leprosy [5]. The research on the archaeological samples refutes the hypotheses about the existence of cross-immunity between tuberculosis and leprosy and suggested an alternative explanation for decline of leprosy from Western Europe. The authors suggested that the immunological changes seen in multi-bacillary leprosy together with socioeconomic impact of the disease may have lead to increased mortality due to tuberculosis which resulted in the decline of leprosy in Western Europe [9]. Despite this long history of epidemiological, clinical and microbiological evidences the exact relationship between tuberculosis and leprosy still remains unclear.

Nepal is one of the five countries where leprosy is endemic and also a high burden country for tuberculosis. In the earlier reports of concomitant occurrence of leprosy and tuberculosis, leprosy was preceded by tuberculosis which is similar to our case presentations [1-3]. This is unlike a case presentation reported from the USA in which a Filipino patient who was undergoing treatment for pulmonary tuberculosis when he was diagnosed to have leprosy also [4]. It is important to note that in our case presentations both the patients were treated for leprosy in the past and had developed complications of leprosy. In our case presentations, concomitant occurrence of tuberculosis may have been a result of steroid treatment for complications of leprosy.

## Conclusion

Treatment with steroid may have decreased the immunity and predisposed the patients to reactivation of tuberculosis. One study has reported that the patients treated with glucocorticoids are at an increased risk of pulmonary tuberculosis [10]. One of our two cases presented with symptoms suggestive of tuberculosis which were of three months duration, and the other patient had a loculated pleural effusion. It was not sure if this was a patient delay or treating physician's delay (health system delay) in our case presentations. Therefore, it becomes imperative for physicians treating leprosy complications with steroids to have a high degree of suspicion to diagnose pulmonary tuberculosis. This infrequent concurrence may not be expected since it is generally believed that cross-immunity exists between TB and leprosy. This should be cautioned in countries like Nepal where leprosy and tuberculosis both are endemic. It is important to screen the patients for TB before initiating long term steroid treatment. Facilities for diagnosis and treatment of both conditions should be made available in the same set up as it may optimize the use of resources. Physicians should recognize that the leprosy patients treated with glucocorticoid may develop tuberculosis. Physicians should screen the patients for TB before initiating long term steroid treatment and undertake necessary periodical investigations to diagnose the concomitant condition.

## Competing interests

The authors of this case presentation declare that they have no financial or non-financial competing interests with regard to the present manuscript.

## Authors' contributions

CTS conceived the study, made substantial contributions to acquisition of data, drafted the initial manuscript, and revised the draft all over the course of submission.

RGM coordinated in designing and drafting the initial draft, and revised the draft all over the course of submission

PVK is a treating clinician, made contributions to acquisition of data, and revised the draft wherever necessary.

All authors read and approved the final manuscript.
